# Multi-omics pan-cancer study of SPTBN2 and its value as a potential therapeutic target in pancreatic cancer

**DOI:** 10.1038/s41598-024-60780-6

**Published:** 2024-04-29

**Authors:** Hongliang Chang, Hong Chen, Taiheng Ma, Kexin Ma, Yi Li, Lida Suo, Xiangnan Liang, Kunyu Jia, Jiahong Ma, Jing Li, Deguang Sun

**Affiliations:** 1 Division of Hepatobiliary and Pancreatic Surgery, Department of General Surgery, The Second Hospital of Dalian Medical University, No. 467 Zhongshan Road, Dalian, 116021 China; 2 Division of Cholelithiasis Minimally Invasive Surgery, Department of General Surgery, Affiliated Zhongshan Hospital of Dalian University, Dalian, 116001 China

**Keywords:** SPTBN2, Pancreatic cancer, Multi-omics, Pan-cancer, Prognosis, Immune, Cancer, Biomarkers

## Abstract

SPTBN2 is a protein-coding gene that is closely related to the development of malignant tumors. However, its prognostic value and biological function in pan-cancer, especially pancreatic cancer (PAAD), have not been reported. In the present study, a novel exploration of the value and potential mechanism of SPTBN2 in PAAD was conducted using multi-omics in the background of pan-cancer. Via various database analysis, up-regulated expression of SPTBN2 was detected in most of the tumor tissues examined. Overexpression of SPTBN2 in PAAD and kidney renal clear cell cancer patients potentially affected overall survival, disease-specific survival, and progression-free interval. In PAAD, SPTBN2 can be used as an independent factor affecting prognosis. Mutations and amplification of SPTBN2 were detected, with abnormal methylation of SPTBN2 affecting its expression and the survival outcome of PAAD patients. Immunoassay results demonstrate that SPTBN2 was a potential biomarker for predicting therapeutic response in PAAD, and may influence the immunotherapy efficacy of PAAD by regulating levels of CD8 + T cells and neutrophil infiltration. Results from an enrichment analysis indicated that SPTBN2 may regulate the development of PAAD via immune pathways. Thus, SPTBN2 is a potential prognostic biomarker and immunotherapy target based on its crucial role in the development of PAAD.

## Introduction

As the incidence and mortality rates of cancer increase each year, not only are individual health and lives adversely affected, but there is an economic burden shouldered by countries worldwide^1,2^. Compared with the three traditional treatments for cancer (surgery, radiotherapy, and chemotherapy), targeted therapy has achieved significant progress in the diagnosis and treatment of various cancers to improve patient prognosis^3–6^. Furthermore, advances in personalized targeted therapy have been enhanced with the development of novel targeted drugs. As a result, the survival of patients with early or advanced cancer has been prolonged. However, there remains an ongoing and urgent need to identify molecular mechanisms and roles of specific cancer genes in order to discover new therapeutic targets. With the development of high-throughput biotechnology, advances in tumor research based on single-omics data have been made. However, studies of single-omics data tend to only reveal tumor-related insights at a single level, rather than characterizing the complex molecular mechanisms that contribute to human tumors^7^. Therefore, in the present study, a multi-omics integrated bioinformatics analysis was conducted with the goal of identifying possible immune target molecules from various public databases. Our focus was to elucidate new targets for treatment of malignant tumors, especially refractory pancreatic cancer (PAAD).

Spectrin b non-erythrocytic 2 (SPTBN2), also termed b-III spectrin, is a protein-coding gene that is closely related to the development of several malignant tumors^8^. SPTBN2 regulates glutamate signaling by stabilizing the glutamate transporter, EAAT4, on the surface of the plasma membrane^9^. Mutations in SPTBN2 can lead to spinocerebellar ataxia, which is characterized by neurodegeneration, progressive dyskinesia, heart rate disorders, and uncoordinated eye movements^10–12^. In addition, high levels of SPTBN2 expression have been significantly associated with poor prognosis in both lung adenocarcinoma (LUAD), breast cancer (BC), bladder urothelial carcinoma (BLCA), colon adenocarcinoma/rectum adenocarcinoma esophageal carcinoma (COADREAD) and uterine corpus endometrial carcinoma (UCEC)^13–16^. In cases of ovarian cancer patients, Feng et al. observed that SPTBN2 mediates an adverse effect to decrease CD4 + T cell infiltration, also leading to a poor prognosis^17^. In addition, in COADREAD and thyroid carcinoma (THCA), SPTBN2 promotes the proliferation and migration ability of tumor cells^18,19^. However, to date, while studies on the role of SPTBN2 in various cancers has been investigated, its role and molecular mechanism(s) in PAAD remain to be investigated in the context of pan-cancer.

In the present study, we investigate the role of SPTBN2 in PAAD at the macro level. Based on multi-omics data, the potential for SPTBN2 to serve as a prognostic biomarker of PAAD and a predictor of immune status and immunotherapy response of the tumor microenvironment (TME) is investigated in PAAD patients. The contribution of DNA methylation in regulating SPTBN2 expression and PAAD progression is also examined. This study also shows for the first time that SPTBN2 may be affected by CD8 + T cells and neutrophils, which would limit its efficacy. Finally, a series of sensitive small molecule drugs are predicted. Taken together, these findings represent a reference for the development of new therapeutic targets and characterization of SPTBN2 as a potential target for clinical treatment of PAAD.

## Results

### SPTBN2 expression

**Figure 1:**
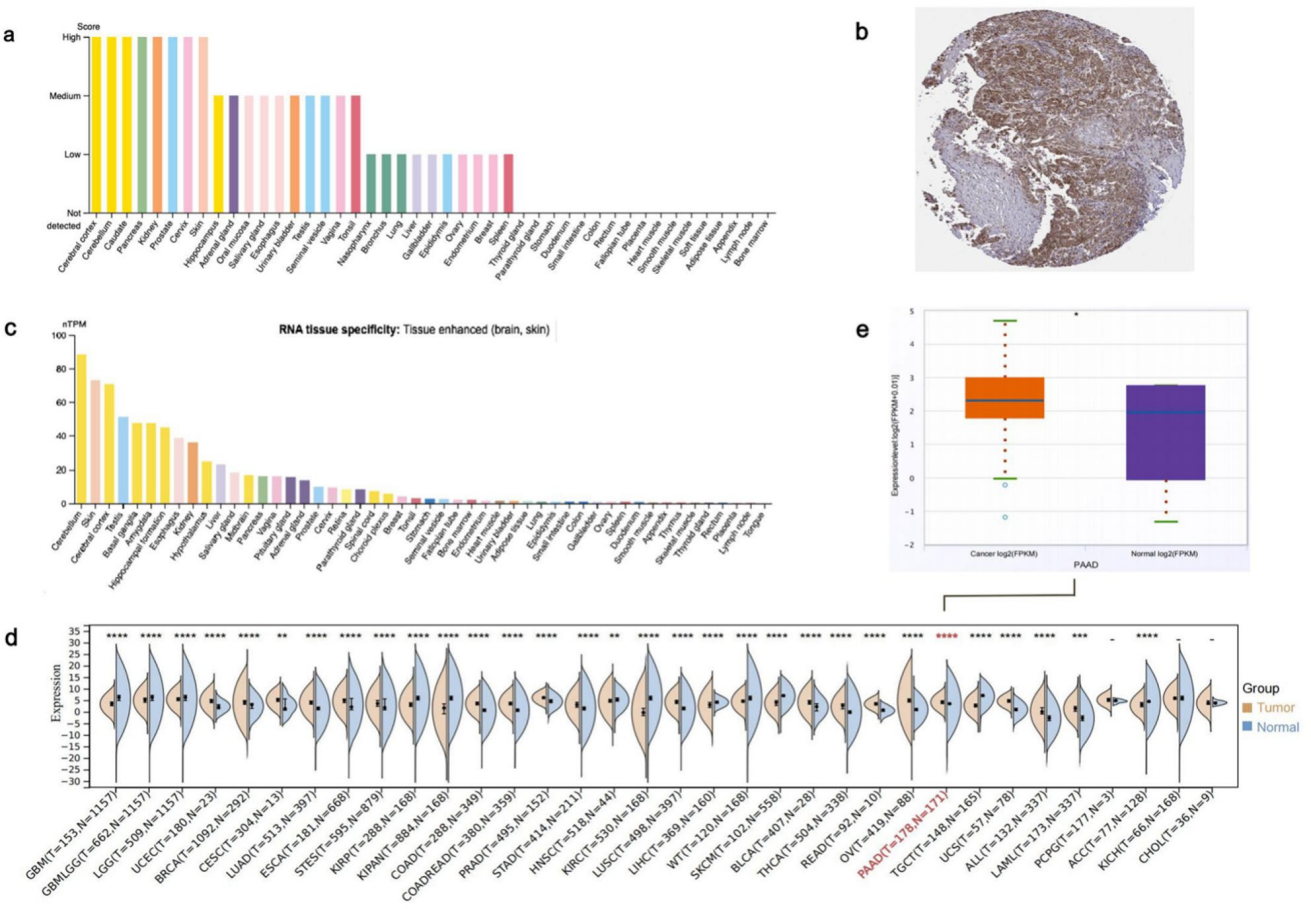
SPTBN2 expression. (**a**) SPTBN2 protein expression. (**b**) SPTBN2 protein expression in PAAD. (**c**) SPTBN2 mRNA expression. (**d**) The expressing levels of SPTBN2 across diverse cancer types. (**e**) Expression analysis of SPTBN2 in PAAD tumor tissues and normal tissues. *p < 0.05, **p < 0.01, ***p < 0.001, ****p < 0.0001.

The expression of SPTBN2 mRNA and protein was investigated in the Human Protein Atlas (HPA) database. Regarding SPTBN2 protein expression, it is higher in the cerebral cortex, cerebellum, caudate, pancreas, kidney, prostate, cervix, and skin (Fig. 1a). Furthermore, immunohistochemical staining showed that SPTBN2 protein was highly expressed in PAAD tumor cells (Fig. 1b). SPTBN2 mRNA was found to be highly expressed in brain and skin, while it is expressed at lower levels in most other normal tissues, including thymus, lung, and colon (Fig. 1c). When expression levels of SPTBN2 were compared between 22 types of tumor tissues and corresponding normal tissues in pan-cancer, statistically significant differences were observed. The highest expression of SPTBN2 was found in PAAD tissues (Fig. 1d–e, *p* < 0.05). In contrast, expression levels of SPTBN2 in normal tissues such as glioblastoma multiforme (GBM), brain lower grade glioma (LGG), kidney renal papillary cell carcinoma (KIRP), head and neck carcinoma (HNSC), kidney renal clear cell carcinoma (KIRC), and liver hepatocellular carcinoma (LIHC) were higher than those in tumor tissues (Fig. 1d, *p* < 0.05).

### Genetic alterations

Some genetic alterations have been shown to affect RNA modifications and to be associated with cancer^20,21^. The cBioPortal online tool was used to analyze possible genetic mutations in SPTBN2, including mutations, structural variants, amplifications, and deep deletions. The most common genetic alterations detected in SPTBN2 were mutations and amplifications. The former were found in UCEC and skin cutaneous melanoma (SKCM), while the latter were found in HNSC and BC (Supplemental Fig. 1a). Similarly, an analysis of the Tumor Immune Evaluation Resource (TIMER) database showed that UCEC (64/531) and SKCM (40/468) had the highest mutation rates of SPTBN2 (Supplemental Fig. 1b). Moreover, missense mutation was the most common type of SPTBN2 alteration (Supplemental Fig. 1c). A somatic mutation analysis further showed somatic cell distribution of the TCGA-PAAD cohort, and that KRAS and TP53 frequency changes were the top two mutations in PAAD patients (Supplemental Fig. 1d, *p* < 0.05).

### Prognostic value of SPTBN2

**Figure 2:**
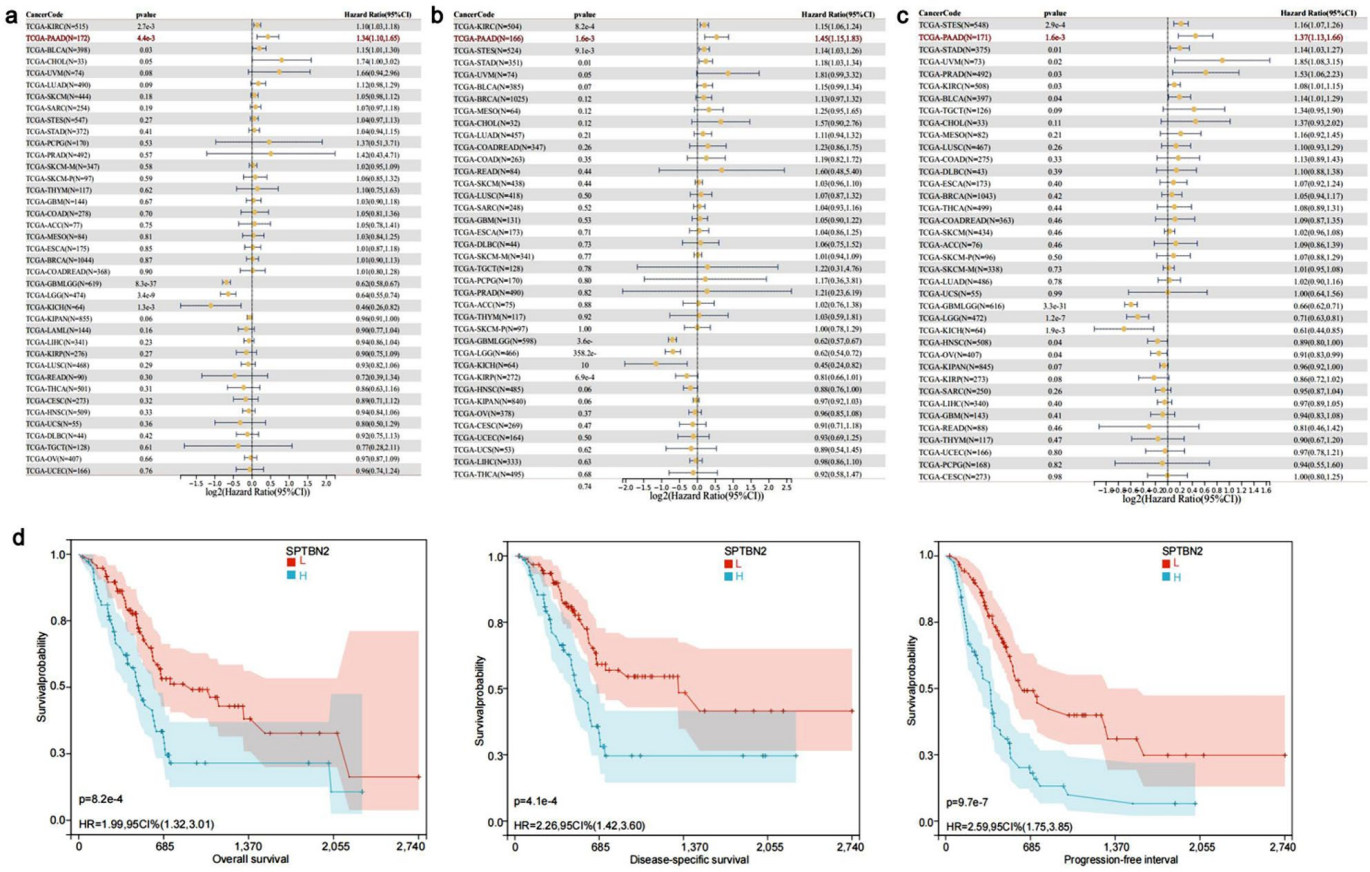
Prognostic value of SPTBN2. Correlations of SPTBN2 expression and (**a**) overall survival (OS), (**b**) disease-specific survival (DSS), and (**c**) progression-free interval (PFI). (**d**) Survival curve.

To better understand the prognostic value of SPTBN2 in pan-cancer, survival analyses were performed based on three prognostic indicators: overall survival (OS), disease-specific survival (DSS), and progression-free interval (PFI). High levels of SPTBN2 expression significantly correlated with OS in KIRC, PAAD, and BLCA, resulting in poor prognosis (Fig. 2a, *p* < 0.05).

For DSS, high expression of SPTBN2 was also associated with poorer survival in most cancers, including KIRC, PAAD, stomach and esophageal carcinoma (STES), and stomach adenocarcinoma (STAD) (Fig. 2b, *p* < 0.05). Based on these results, SPTBN2 is considered a high-risk gene.

Cox regression analysis demonstrated that SPTBN2 is significantly correlated with PFI in STES, PAAD, STAD, uveal melanoma (UVM), prostate adenocarcinoma (PRAD), KIRC, and BLCA (Fig. 2c, *p* < 0.05).

Kaplan–Meier survival curves of PAAD patients showed that high expression of SPTBN2 correlated with poor OS, DSS, and PFI (Fig. 2d, *p* < 0.05), which was consistent with the previous Cox regression results.

Taken together, these results indicate that expression of SPTBN2 is associated with the prognosis of multiple cancers. In particular, SPTBN2 significantly affects OS, DSS, and PFI of PAAD patients, and may represent a valuable biomarker for prognosis.

### Relationship between SPTBN2 and clinical indicators

**Figure 3:**
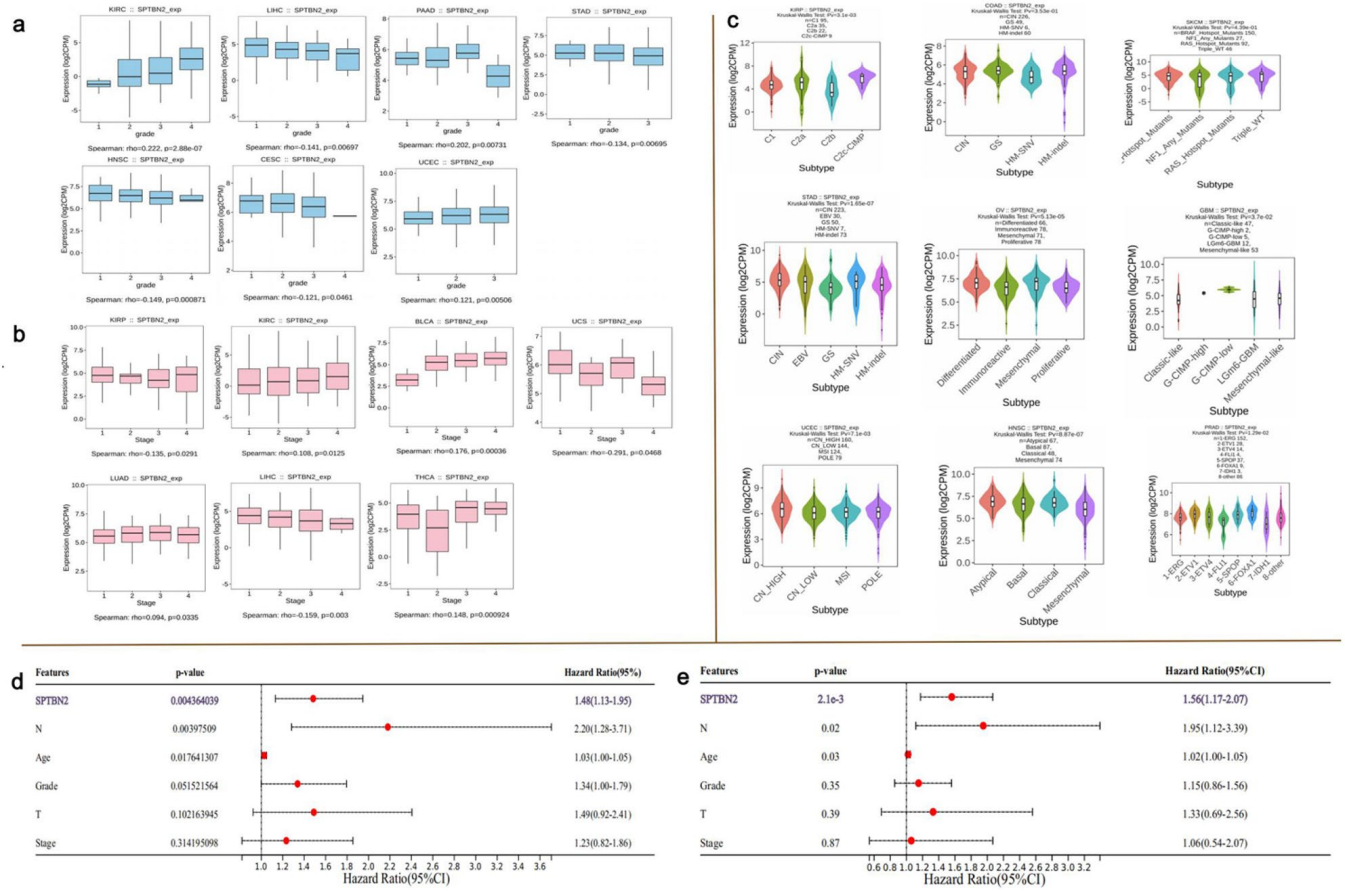
Relationship between SPTBN2 and clinical indicators. (**a**) Relationship between SPTBN2 and grade. (**b**) Relationship between SPTBN2 and stage. (**c**) Relationship between SPTBN2 and subtype. (**d**) Results of Univariate Cox regression analysis. (**e**) Results of multivariable Cox regression analysis.

To further characterize SPTBN2 as a potential prognostic marker, expression of SPTBN2 in different pathological stages, histological grades, and subtypes was examined. SPTBN2 overexpression significantly correlated with high grade progression of UCEC, high stages of BLCA, and both high grades and stages of KIRC (Fig. 3a, b, *p* < 0.05). In addition, SPTBN2 correlated with grades of cervical squamous cell carcinoma and endocervical adenocarcinoma (CESC), LIHC, STAD, HNSC, and PAAD (Fig. 3a, *p* < 0.05); while it significantly correlated with stages of KIRP, uterine carcinosarcoma, LUAD, LIHC, and THCA (Fig. 3b, *p* < 0.05). SPTBN2 was also found to be closely related to certain tumor subtypes, including KIRP, colon adenocarcinoma (COAD), and SKCM (Fig. 3c, *p* < 0.05).

Considering the association between SPTBN2 and survival outcomes in patients with PAAD, we further evaluated whether SPTBN2 is an independent prognostic factor for PAAD. Univariate Cox regression analysis showed that SPTBN2 could predict the prognosis of PAAD patients (Fig. 3d, *p* < 0.05). In a subsequent multivariable Cox regression analysis, SPTBN2 remained statistically significant (Fig. 3e, *p* < 0.05), indicating that SPTBN2 was an independent prognostic factor for PAAD.

### DNA methylation analysis of SPTBN2

**Figure 4:**
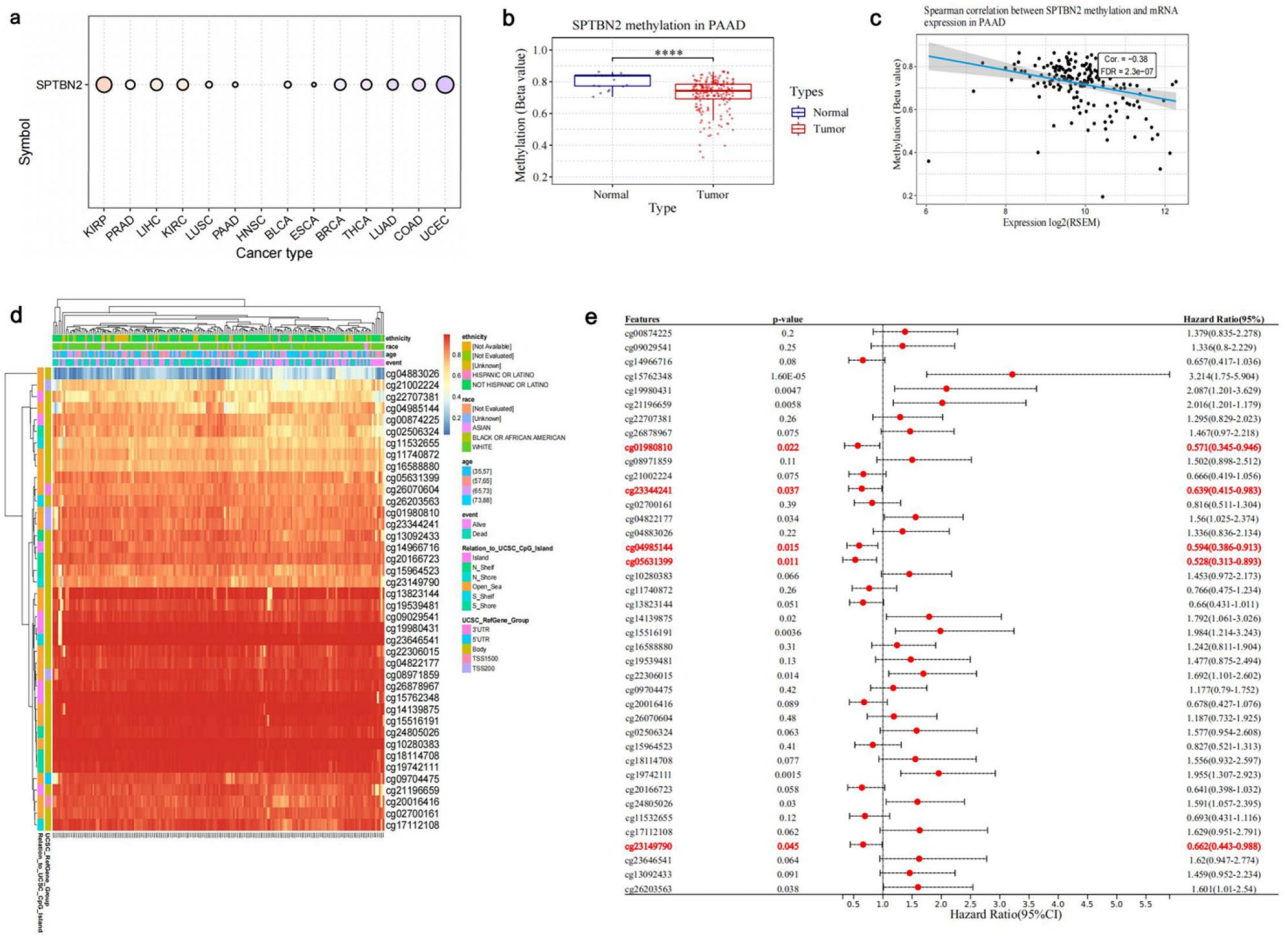
DNA methylation analysis of SPTBN2. (**a**) The SPTBN2 DNA methylation levels in pan-cancer. (**b**) The SPTBN2 DNA methylation levels in PAAD. (**c**) Correlation between the mRNA expression and DNA methylation levels. (**d**) The heatmap of DNA methylation. (**e**) The prognostic value of DNA methylation of SPTBN2 in PAAD with different CpG sites. *p < 0.05, **p < 0.01, ***p < 0.001, ****p < 0.0001.

DNA methylation is an important post-transcriptional epigenetic regulator and plays an important role in tumorigenesis^22,23^. Methylation levels of SPTBN2 were found to differ significantly between KIRP, PRAD, LIHC, KIRC, Lung squamous cell carcinoma (LUSC), PAAD, HNSC, BLCA, Esophageal carcinoma (ESCA), BC, THCA, LUAD, COAD, and UCEC and their corresponding normal tissues (Fig. 4a, *p* < 0.05). In particular, the DNA methylation level of SPTBN2 in PAAD tumor tissues compared with normal samples was significantly reduced (Fig. 4b, *p* < 0.05). Furthermore, mRNA expression of SPTBN2 negatively correlated with its methylation level (Fig. 4c, *p* < 0.05). In Fig. 4d, the methylation map of SPTBN2 in PAAD contains a total of 40 CpG sites. A survival analysis suggests that 15 CpGs are associated with the prognosis of PAAD patients, among which five sites play a protective role, including cg01980810, cg23344241, cg04985144, cg05631399, and cg23149790 (Fig. 4e, *p* < 0.05). Overall, these results suggest that DNA methylation of SPTBN2 may contribute to the development of PAAD and is closely related to the prognosis of PAAD patients.

### Immunoinfiltration analysis of SPTBN2

**Figure 5:**
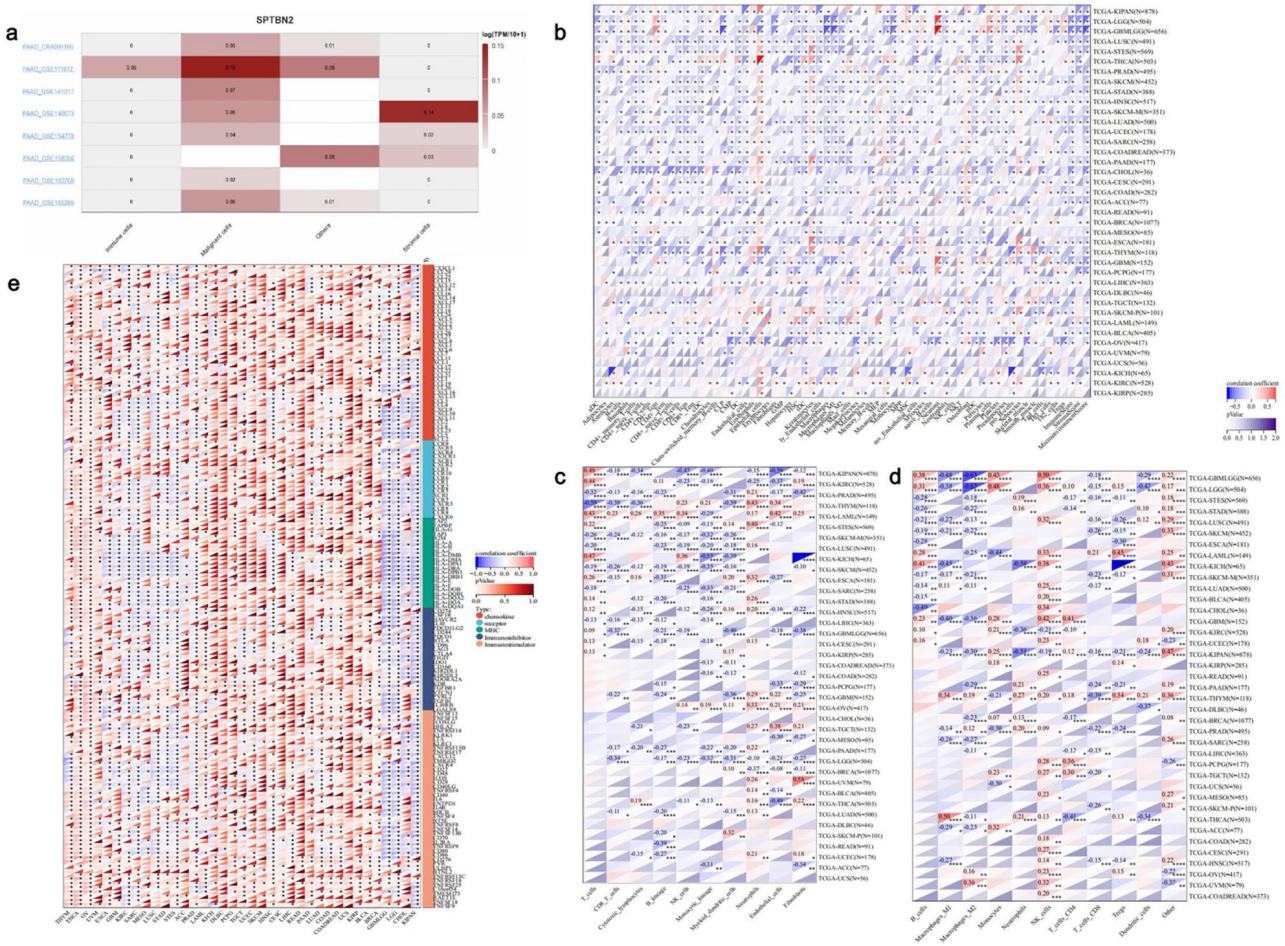
Immunoinfiltration analysis of SPTBN2. (**a**) The cell types and their distribution. (**b**) The xCELL algorithm. (**c**) The MCPcounter algorithm. (**d**) The QUANTISEQ algorithm. (**e**) Relationship between SPTBN2 and immunomodulatory genes. *p < 0.05, **p < 0.01, ***p < 0.001, ****p < 0.0001.

As an integral part of the TME, tumor-infiltrating immune cells play a crucial role in tumor progression and development^24–26^. We performed a single-cell analysis and found that SPTBN2 is significantly enriched in PAAD tumor cells (Fig. 5a). To further detect possible associations between different immune cells and SPTBN2 levels in different tumor types in TCGA, the xCELL, MCPcounter, and QUANTISEQ algorithms were applied. The results from xCELL suggest that expression of SPTBN2 negatively correlates with the infiltration levels of most immune cells in ovarian serous cystadenocarcinoma and cholangiocarcinoma (Fig. 5b, *p* < 0.05). In addition, expression of SPTBN2 correlates with the proportion of immune and stromal cells in each tumor, suggesting that SPTBN2 levels are closely correlated with immune activation in a variety of tumors such as PAAD (Fig. 5b, p < 0.05). Results from the MCPcounter algorithm showed that SPTBN2 negatively correlates with CD8 + T cells, cytotoxic lymphocytes, B lineage cells, monocytic lineage cells, and myeloid dendritic cells; while it positively correlates with neutrophils, in PAAD (Fig. 5c, *p* < 0.05). When the differences and correlations observed were further analyzed using the QUANTISEQ algorithm, expression of SPTBN2 was found to correlate with the degree of immune cell infiltration (Fig. 5d, *p* < 0.05). In addition, SPTBN2 levels negatively correlated with M2 macrophages, CD8 + T cells, and regulatory T cells of PAAD; while being positively correlated with neutrophils (Fig. 5d, *p* < 0.05). Correlations between SPTBN2 and immune cells that were consistent across two or three algorithms were considered meaningful. Notably, SPTBN2 negatively correlated with the infiltration level of CD8 + T cells and positively correlated with neutrophils. Furthermore, strong negative associations were observed between SPTBN2 and multiple immunomodulatory genes (Fig. 5e, *p* < 0.05). For example, a few key monocyte/macrophage chemokines (i.e., CCL15, CXCL12, etc.) were found to be down-regulated in the SPTBN2 high-expression group, thereby inhibiting the inflammatory response and monocyte/macrophage phagocytosis in PAAD. In addition, up-regulation of SPTBN2 in PAAD was associated with increased expression of chemokine receptors (CXCR5, CCR10), major histocompatibility complex (MHC) molecules (TAP2, TAPBP), immunosuppressive molecules (TGFB1), and immunostimulatory molecules (TNFSF13, CD40, NT5E, and CD276). Taken together, these results suggest that expression of SPTBN2 regulates the recruitment of immune cells, and may affect the sensitivity of PAAD patients to immunotherapy.

### SPTBN2 is associated with immunotherapy response

**Figure 6:**
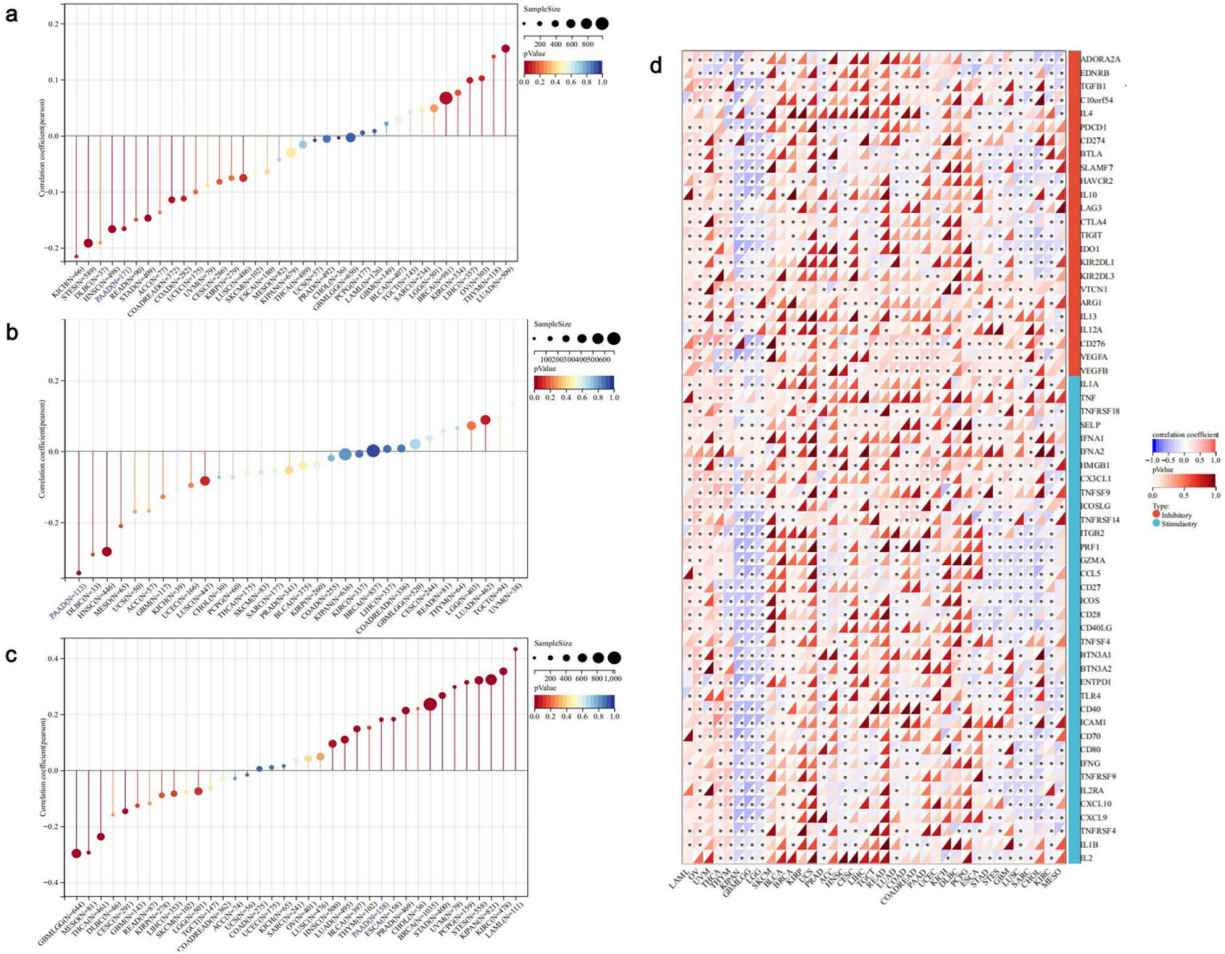
SPTBN2 is associated with immunotherapy response. Relationship between SPTBN2 and (**a**) TMB, (**b**) NEO, (**c**) LOH, and (**d**) ICP genes. *p < 0.05, **p < 0.01, ***p < 0.001, ****p < 0.0001.

To further evaluates the potential value of SPTBN2 in cancer immunotherapy, tumor mutation burden (TMB) scores were examined. Tumor cells with high TMB scores have strong antigenicity and a greater number of neoantigens, thus promoting immune cell infiltration^27,28^. The results obtained indicate that SPTBN2 positively correlates with TMB in LUAD and BC, yet inversely correlates with TMB in COADREAD, STES, STAD, HNSC, and PAAD (Fig. 6a, *p* < 0.05). Next, we explored a possible association between SPTBN2 and neoantigen (NEO). A statistically significant negative association was observed in HNSC and PAAD (Fig. 6b, *p* < 0.05). Conversely, the expression level of SPTBN2 was found to positively correlate with loss of heterozygosity (LOH) in PAAD (Fig. 6c, *p* < 0.05).

Tumor immunotherapy controls and eliminates tumors by restoring and maintaining the tumor immune cycle and restoring normal anti-tumor immune responses. These treatments can include immune checkpoint-blocking (ICB) and cell therapies. The effectiveness of ICB therapy depends not only on immune cell penetration and TMB, but also on immune checkpoint (ICP)^29^. SPTBN2 expression was found to negatively correlate with almost all of the ICP genes in several cancers, including GBM and LGG. Furthermore, in PAAD, SPTBN2 significantly positively correlated with VEGFB, TGFB, CD276, and VEGF; while being significantly negatively correlated with IL1B, TLR4, CD40LG, and CCL5 (Fig. 6d, *p* < 0.05). These results suggest that SPTBN2 influences the sensitivity of PAAD to ICB therapy, and may be a more effective biomarker to predict efficacy of immunotherapy.

### SPTBN2 and tumor stemness

Cancer progression involves progressive loss of differentiated phenotypes and acquisition of progenitor and stem-cell-like features^30^. Tumor stemness is associated with a suppressed immune response, higher intratumoral heterogeneity, and marked deterioration of most cancers^30,31^. When we further investigated the relationship between SPTBN2 expression and tumor stem cell score, a significant positive correlation was observed between SPTBN2 expression and both DNAss (Supplemental Fig. 2a) and EREG-METHss (Supplemental Fig. 2b) scores in most malignancies, but especially in PAAD, CESC, and STES (*p* < 0.05). These results suggest that tumors characterized by high levels of SPTBN2 expression may generally contribute to stemness maintenance.

### Possible mechanism of action of SPTBN2 in PAAD

**Figure 7:**
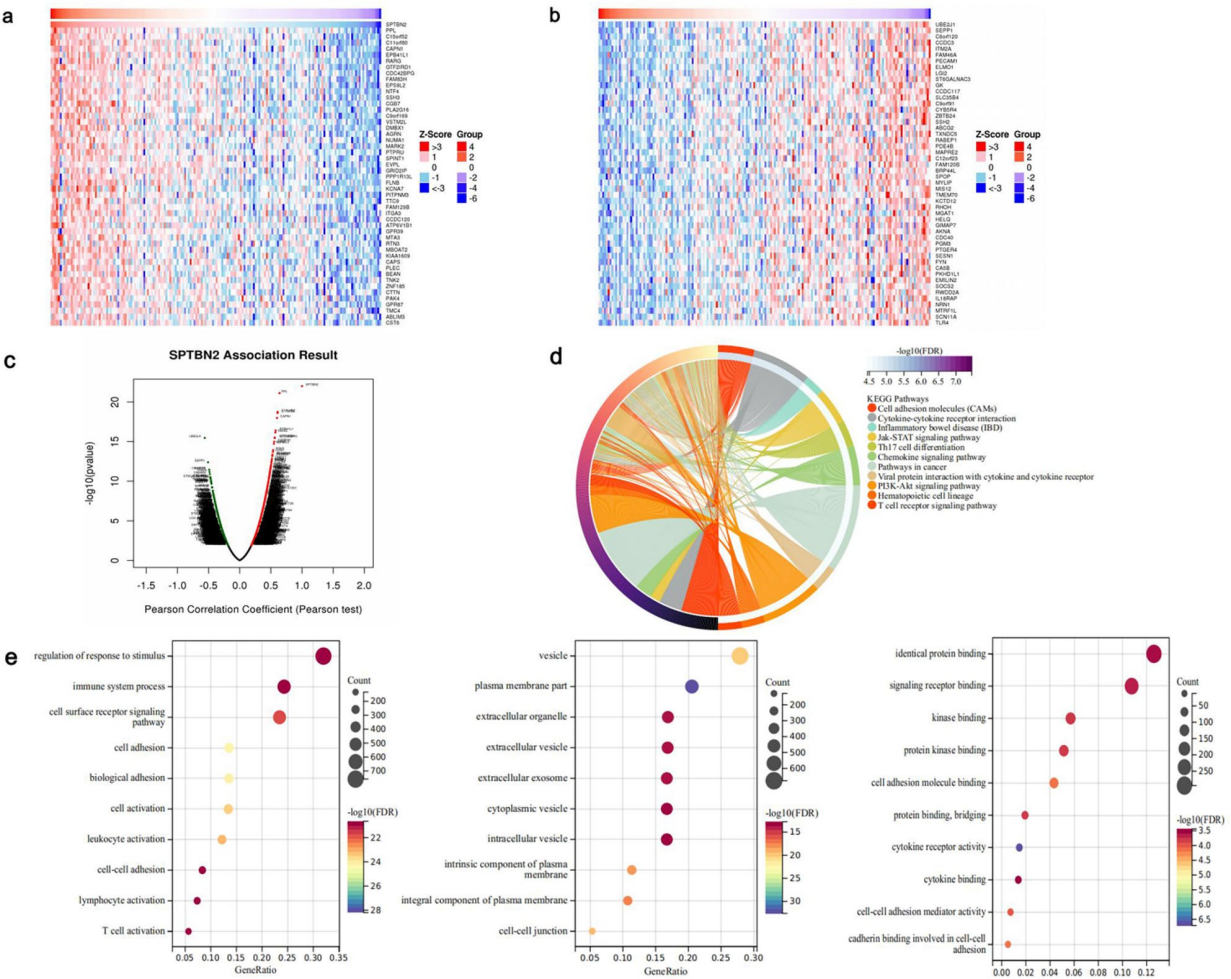
Possible mechanism of action of SPTBN2 in PAAD. (**a**) The heatmap of the top 50 genes with a positive correlation with SPTBN2. (**b**) The heatmap of the top 50 genes with a negative correlation with SPTBN2. (**c**) A volcano map of the SPTBN2 and its co-expressed genes. (**d**) Kyoto Encyclopedia of Genes and Genomes (KEGG) pathway analysis. (**e**) Gene Ontology (GO) analysis. *p < 0.05, **p < 0.01, ***p < 0.001, ****p < 0.0001.

Genes co-expressed with SPTBN2 were explored using the LinkedOmics database. A total of 1445 positively and 1580 negatively related genes were identified (false discovery rate (FDR) < 0.01). Expression heat maps of the top 50 positively and negatively correlated genes are shown in Fig. 7a, b, respectively. Volcano maps include all of the co-expressed genes (Fig. 7c). An analysis of the Kyoto Encyclopedia of Genes and Genomes (KEGG) pathway for all of the co-expressed genes shows that the target genes are mainly associated with: “Pathways in cancers”, “Hematopoietic cell lineage”, and “P13K-Akt signaling pathway” (Fig. 7d, FDR < 0.05). Meanwhile, the gene ontology (GO) analysis showed that most of the co-expressed genes are related to immune pathways (Fig. 7e, FDR < 0.05).

**Figure 8:**
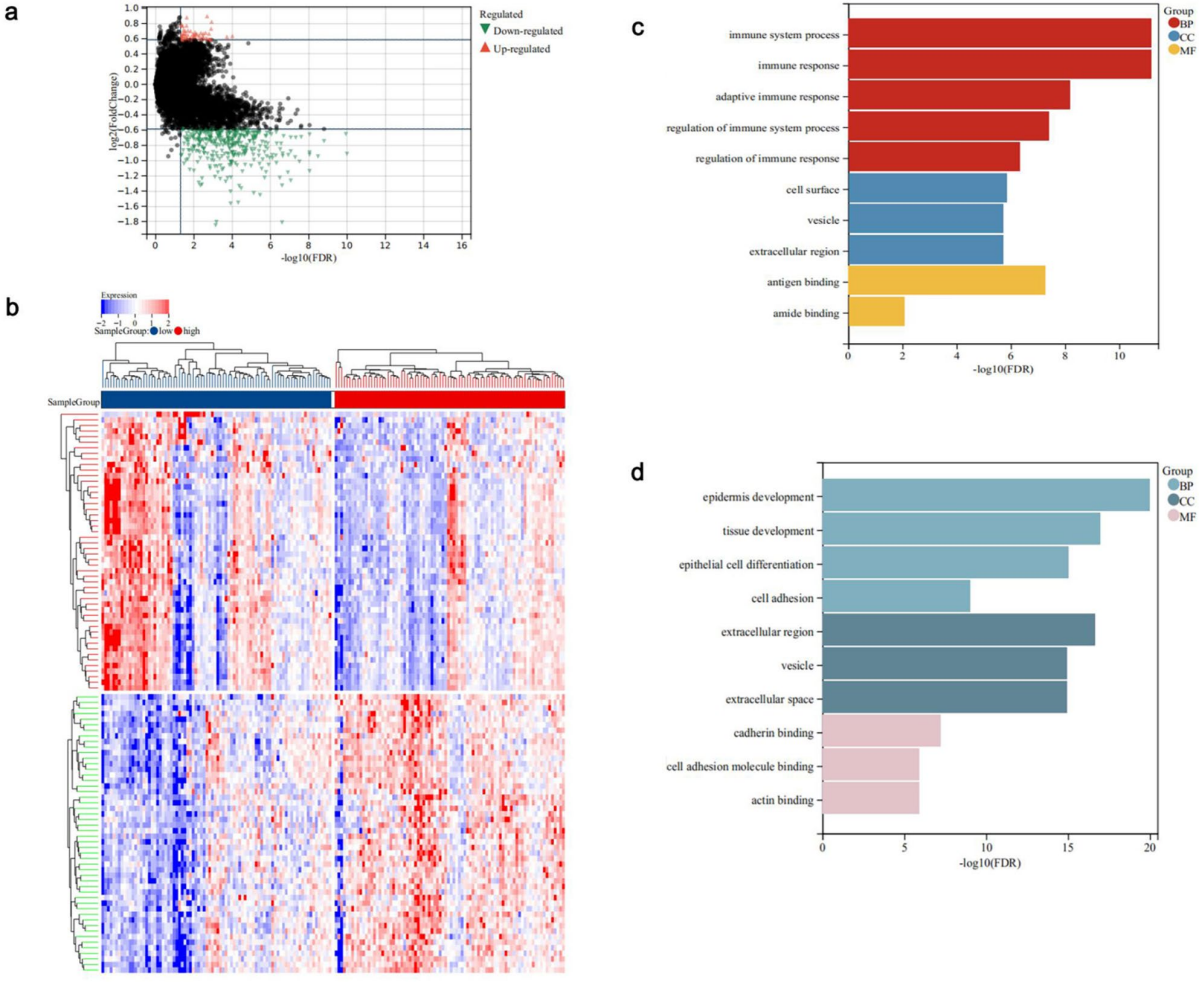
Possible mechanism of action of SPTBN2 in PAAD. (**a**) A volcano map. (**b**) A heatmap. (**c**) Enrichment analysis for GO term of up-regulated genes. (**d**) Enrichment analysis for GO term of down-regulated genes.

When we analyzed differentially expressed genes (DEGs) between the high and low expression groups in PAAD patients, a total of 430 DEGs were identified in the low expression group compared with the high expression group (58 up-regulated genes, 372 down-regulated genes) (Fig. 8a, b, fold change (FC) > 1.5, FDR < 0.05). Compared with the enrichment pathways of the down-regulated genes, the up-regulated genes were found to be significantly enriched in immune pathways such as “immune system process”, “immune response”, and “adaptive immune response” (Fig. 8c, d, FDR < 0.05). These results suggest that SPTBN2 may contribute to immune regulation to affect the development of PAAD.

### Prediction of drug sensitivity based on SPTBN2 expression

Finally, to fully explore the value of SPTBNT2 as a novel immune target in PAAD, we predicted drug sensitivity based on SPTBNT2 expression (Supplemental Fig. 3). Using the Genomics of Drug Sensitivity in Cancer dataset, an investigation of a possible correlation between SPTBN2 levels and drug sensitivity suggested that Vorinostat (a HDAC inhibitor) inversely correlates with SPTBN2 expression (Supplemental Fig. 3a). Conversely, Lapatinib (a tyrosine kinase inhibitor) positively correlated with SPTBN2 expression (Supplemental Fig. 3a). When the Cancer Therapeutics Response Portal dataset was used, a correlation between SPTBN2 levels and drug sensitivity showed that Afatinib (a tyrosine kinase inhibitor) and Belinostat (a HDAC inhibitor) negatively correlated with SPTBN2 expression, yet positively correlated with Staurosporine (a PKC inhibitor) (Supplemental Fig. 3b). Overall, these results demonstrate the immunotherapeutic value of SPTBN2 in PAAD. Moreover, a series of targeted and small molecule drugs with good therapeutic effects are predicted, and this provides options for immunotherapy targeting SPTBN2 in PAAD.

## Discussion

SPTBN2 is located on chromosome 11 and encodes spectrin beta, non-erythrocytic 2, also known as beta-III spectrin^32^. Spectrin is the main component of the cell membrane cytoskeleton and consists of two alpha subunits and two beta subunits^33–35^. Mutations in SPTBN2 can lead to spinocerebellar ataxia^10–12^. In addition, SPTBN2 has been associated with BC, UCEC, BLCA, and COADREAD^13,36,37^. SPTBN2 also plays a considerable role in both tumor genesis and metastasis^13,36,37^. However, analysis of a single type of cancer limits our ability to fully understand the multifaceted nature of a target gene and its underlying mechanisms. Multi-omics analysis can provide a more comprehensive understanding of the molecular mechanisms of tumor development, while also facilitating the discovery of new biomarkers and therapeutic targets. Thus, multi-omics is of great significance for the early diagnosis and treatment of cancers^38^. In the present study, by analyzing the multi-omics data, including SPTBN2 expression levels, genetic alterations, prognostic value, DNA methylation, tumor immunity, and mechanisms of action, the value of SPTBN2 in pan-cancer was comprehensively and systematically explored, in particular the role of SPTBN2 in PAAD.

SPTBN2 was found to be abnormally expressed at high levels in different tumor types, including PAAD, and at low levels in KIRC tissues. Zhang et al. previously determined that SPTBN2 expression is significantly upregulated in patients with COADREAD^37^. Consistent with this result, SPTBN2 expression appeared to be higher in COADREAD tumor tissues than in normal tissues in the present study. Similarly, SPTBN2 was found to be expressed at a high level in BC, UCEC, BLCA and LUAD tumors, and low expression in LGG tumors, also consistent with previous findings^13,14,16,39^. An analysis of genetic alterations in SPTBN2 showed that mutations and amplification events were most common in UCEC, SKCM, HNSC, and BC. In PAAD, patients with high levels of SPTBN2 expression have a higher proportion of KRAS and TP53 mutations, as well as a poorer prognosis, compared with patients with low levels of SPTBN2 expression. This result is consistent with previous findings that PAAD patients with TP53 and KRAS mutations exhibit worse survival rates^40–42^. Survival analysis showed that high expression of SPTBN2 was significantly associated with poor prognosis in BLCA, consistent with previous findings^13^. Furthermore, the expression levels of SPTBN2 in PAAD and KIRC correlate with OS, DSS, and PFI, and these expression levels were a negative prognostic factor. It is worth noting that expression of SPTBN2 significantly correlated with the stage and grade of KIRC and LIHC, and also closely correlated with PAAD grade. These results may help guide the clinical selection of drugs and treatment regimens. Considering the prognostic value of SPTBN2 in PAAD, we further explored the role of SPTBN2 by univariate and multivariable Cox regression analyses. The results obtained suggest that SPTBN2 is an independent prognostic factor of PAAD, and high expression of SPTBN2 is closely related to poor prognosis. Overall, these results suggest that SPTBN2 plays an important role in the pathogenesis and prognosis of tumors, and is an independent biomarker of PAAD.

DNA methylation plays a crucial role in tumor development. Up- or down-regulation of DNA methylation can affect the expression of tumor genes, and thus, tumor progression^43,44^. In most tumors, we observed a potential link between methylation levels and mRNA levels of SPTBN2. In addition, compared with normal tissues, methylation levels of SPTBN2 were significantly lower in PAAD tumor tissues, and they significantly negatively correlated with mRNA levels^45,46^. Given that high expression of SPTBN2 is associated with poorer survival outcomes, and that methylation levels of SPTBN2 negatively correlate with mRNA levels, thus that high levels of methylation should be associated with better survival. In fact, high methylation levels at cg01980810, cg23344241, cg04985144, cg05631399, and cg23149790 were associated with better outcomes in patients with PAAD. Paradoxically, we observed that some CpGs were associated with poor outcomes in PAAD patients, and possible factors need to be further explored. However, the present results indicate that abnormal SPTBN2 promoter hypomethylation may be a major genomic driver of increased SPTBN2 expression in PAAD, and blocking epigenetic level abnormal SPTBN2 expression may represent a potential therapeutic strategy to reverse the occurrence of PAAD.

In recent years, the TME has been recognized as having a key role in tumor initiation, progression, metastasis, and immunotherapy response^47–50^. A single-cell analysis showed that SPTBN2 is highly expressed in tumor cells, which suggests that SPTBN2 may promote cancer. Consistent with previous findings, SPTBN2 was inversely associated with most levels of immune cell infiltration in LGG^39^. In addition, SPTBN2 was found to be significantly positively correlated with neutrophil infiltration levels, yet negatively correlated with CD8 + T cells. Neutrophils escort circulating tumor cells to achieve cell cycle progression and tumor progression. They also act as immunosuppressive cells associated with immune resistance^51,52^. Cancer-associated neutrophilia and tumor infiltration of neutrophils are significant markers of poor outcome in many cancers^53^. Therefore, SPTBN2 may participate in neutrophil-mediated immunosuppression to achieve a tumor promoting effect in PAAD. In addition, CD8 + T cells are the main effector cells that kill tumor cells and they also play an important role in tumor immune surveillance^54,55^. Previous studies have reported that KRAS-mutated cancer cells contribute to an immunosuppressive environment by regulating immune cell behavior in PAAD^56^. For example, KRAS-mutated cancer cells secrete granulocyte macrophages and colony-stimulating factor, and this can induce myeloid-derived suppressor cell trafficking and inhibit the behavior of CD8 + T cells^57^. KRAS activation also blocks the antigen presentation pathway to facilitate evasion of CD8 + T cells^58^. In the present study, high levels of SPTBN2 expression were found to be associated with a high frequency of KRAS mutations. Based on these results, we speculate that SPTBN2 overexpression and KRAS activation may interact to promote tumor growth and inhibit the penetration of CD8 + T cells in PAAD. Thus, SPTBN2 may be involved in the regulation of neutrophils and CD8 + T cells to promote PAAD progression. At the same time, our results show that SPTBN2 is significantly correlated with TMB, NEO, and LOH of PAAD. Furthermore, a significant positive correlation was observed between SPTBN2 and inhibitory immune checkpoints such as VEGFB, TGFB, CD276, and VEGF; while a significant negative correlation was observed between SPTBN2 and stimulant immune checkpoints such as IL1B, TLR4, CD40LG, and CCL5. Combined with the role of SPTBN2 in the TME, we reasonably speculate that SPTBN2 may be a potential predictive biomarker of immunotherapy response in patients with malignant tumors such as PAAD. Thus, targeting inhibition of SPTBN2 may change the immune microenvironment and improve the efficacy of immunotherapy.

Considering the cancer-promoting effect of SPTBN2 in PAAD, we conducted a preliminary exploration of the biological function(s) of SPTBN2 at the genetic level. The KEGG enrichment analysis performed suggests that SPTBN2 co-expression genes are mainly concentrated in pathways associated with cancer development, including “Pathways in cancers”, “Hematopoietic cell lineage”, and “P13K-Akt signaling pathway”. A GO analysis further suggested that SPTBN2 is related to immune pathways and may play a complex immunomodulatory role in the TME. When DEGs were identified based on high versus low expression levels of SPTBN2 in PAAD, the enrichment analysis showed that the up-regulated genes were significantly enriched in immune related pathways, such as “immune system process”, compared with the down-regulated genes. These findings confirm that SPTBN2 may play a complex immunomodulatory role in the PAAD microenvironment. Another important finding was that SPTBN2 expression correlated with sensitivity to multiple drugs, including Vorinostat (HDAC inhibitor), Afatinib (tyrosine kinase inhibitor), Belinostat (HDAC inhibitor), and Lapatinib (tyrosinase inhibitor)^59–61^. Taken together, these findings provide strong evidence that targeting of SPTBN2 in PAAD is warranted and should be further studied. Correspondingly, we predicted a series of targeted small molecule drugs with good therapeutic effects.

There were limitations associated with the present study. First, despite exploring the expression and prognostic value of SPTBN2 in different cancer types, there are no clinical data to validate these findings. Second, further studies are needed to confirm the possible mechanism of action of SPTBN2 in PAAD that we propose based on our results. Finally, although this study is the first to show that SPTBN2 affects the progression and efficacy of PAAD by regulating CD8 + T cells and neutrophils, further validation is needed from both in vitro and in vivo studies.

## Conclusions

In summary, we innovatively performed a multi-omics pan-cancer analysis of SPTBN2 which provides strong evidence for the prognostic and immunological value of SPTBN2 in various tumors. Specifically, SPTBN2 is not only a potential prognostic indicator for PAAD patients, but also correlates with immune cell infiltration levels, immune checkpoint expression, and immunotherapy response, and is a potential immunotherapeutic biomarker for PAAD that can be used to select PAAD patients who may benefit from ICB therapy. Importantly, this study is the first to show that SPTBN2 may influence the progression and outcome of PAAD patients by regulating the level of neutrophil or CD8T + cell infiltration. In addition, DNA methylation of SPTBN2 regulates its mRNA expression in PAAD, affecting patient survival outcomes.

In conclusion, our study comprehensively explores the value of SPTBN2 in PAAD and its molecular mechanism, providing a basis for future immunotherapy and precision medicine.

## Materials and methods

### Analysis of gene and protein expression of SPTBN2

We downloaded the standardized pan-cancer dataset, from the UCSC database, consisting of 10,535 samples and 60,499 genes (https://xenabrowser.net). The differences in SPTBN2 expression between various tumor tissues and their corresponding normal tissues were analyzed using R (version 3.6.4). The Human Protein Atlas (HPA) database was used to analyze the expression of SPTBN2 in different tissues (https://www.proteinatlas.org). The integrated repository portal for tumor-immune system interactions (TISIDB) was used to analyze the expression of SPTBN2 in different tumor stages, grades, and subtypes (http://cis.hku.hk/TISIDB/). *P*-values less than 0.05 were considered significant.

### Analysis of genetic alterations

The genetically altered signature of SPTBN2 in various cancers was obtained from the cBioPortal database. The rate of genetic mutations in SPTBN2 in various tumors was explored using the “Gene Mutation” module of the Tumor Immune Evaluation Resource 2.0 (TIMER2) website (http://timer.cistrome.org). Somatic mutation data were derived from the TCGA dataset. A waterfall map reflects the top 15 genes having the highest mutation frequency based on SPTBN2 expression in PAAD. *P*-values less than 0.05 were considered significant.

### Prognostic analysis

Clinical data were downloaded from the TCGA database and subjected to univariate Cox regression analysis to analyze the association between SPTBN2 expression and pan-cancer prognosis. Difference in prognosis between SPTBN2 expression and PAAD patients was also assessed using the R packet “survival”. Univariate and multivariable Cox regression analyses were performed to explore whether SPTBN2 is an independent predictor of prognosis in patients with PAAD. *P*-values less than 0.05 were considered significant.

### DNA methylation analysis

The relationship between SPTBN2 mRNA levels and DNA methylation levels were evaluated using the GSCA database. The “Gene visualization” module of the MethSurv database was used to generate a methylation map of SPTBN2 in PAAD (https://biit.cs.ut.ee/methsurv/). The impact of DNA methylation of each CpG sequence in SPTBN2 in relation to PAAD patient survival was also examined. *P*-values less than 0.05 were considered significant.

### Immunological analysis

QUANTISEQ, MCPcounter, and xCELL algorithms were used to score infiltration of B cells, CD4 + T cells, CD8 + T cells, neutrophils, macrophages, dendritic cells, and other immune cells in each tumor type based on gene expression. Possible correlations between SPTBN2 and marker genes of five immune pathways (chemokine, receptor, major histocompatibility complex (MHC), immunoinhibitor, and immunostimulator) were also examined. *P*-values less than 0.05 were considered significant.

### Immune response analysis

Tumor mutation burden (TMB), loss of heterozygosity (LOH), and neoantigen (NEO) were evaluated. Correlations between TMB, LOH, NEO, and expression of SPTBN2 were then examined. Relationships between immune checkpoint (ICP) genes and SPTBN2 expression were also examined. *P*-values less than 0.05 were considered significant.

### Tumor cell stemness

Two tumor cell stemness scores, DNAss and EREG-METHss, were calculated based on methylation signatures. Possible associations between SPTBN2 and these stemness scores were analyzed. *P*-values less than 0.05 were considered significant.

### Possible mechanism of action of SPTBN2

LinkedOmics was used to explore genes co-expressed with SPTBN2. The results are presented in volcanic maps and heat maps (https://www.linkedomics.org/login.php). Gene ontology (GO) and Kyoto Encyclopedia of Genes and Genomes (KEGG) analyses were subsequently performed using the R package “clusterProfiler” (version 3.14.3) to obtain gene enrichment results. *P* < 0.05 and false discovery rate (FDR) < 0.05 were considered significant.

According to the median level of SPTBN2 expression, PAAD patients were divided into a high expression group and a low expression group. The “limma” R package was used to identify differentially expressed genes (DEGs) between these two groups (Fold change (FC) > 1.5, FDR < 0.05). KEGG and GO analyses were then performed using the R package “clusterProfiler” (version 3.14.3) to obtain gene enrichment results. *P* < 0.05 and FDR < 0.05 were considered significant.

### Immunotherapy response and sensitive drug predictions based on SPTBN2 expression

The GSCA Lite online tool was used to explore the association between SPTBN2 expression and patient sensitivity to current PAAD-targeted drugs (https://guolab.wchscu.cn/GSCA/).

### Supplementary Information

Supplementary Figures.

## Data Availability

All data generated or analysed during this study are included in this published article (and its Supplementary Information files).
