# Supplementary material for: Multi-omics pan-cancer study of SPTBN2 and its value as a potential therapeutic target in pancreatic cancer
Source: Sci Rep. 2024 Apr 29;14:9764. doi: 10.1038/s41598-024-60780-6 (PMC11059406; doi:10.1038/s41598-024-60780-6)
Supplement: Supplementary file 1 — Supplementary Figures. [file 41598_2024_60780_MOESM1_ESM.pdf]

Multi-omics pan-cancer study of SPTBN2 and its value as a potential therapeutic target in pancreatic cancer

Hongliang Chang [2,†], Hong Chen [1,†], Taiheng Ma [1,†], Kexin Ma [1], Yi Li [1], Lida Suo [1], Xiangnan Liang [1], Kunyu Jia [1], Jiahong Ma [1], Jing Li [1], Deguang Sun [1,*]

[1] Division of Hepatobiliary and Pancreatic Surgery, Department of General Surgery, The Second Hospital of Dalian Medical University, Dalian, 116021, China

[2] Division of Cholelithiasis Minimally Invasive Surgery, Department of General Surgery, Affiliated Zhongshan Hospital of Dalian University, Dalian, 116001, China

[†]These authors contributed equally to this work.

*** Corresponding author:**

Deguang Sun, MD

Division of Hepatobiliary and Pancreatic Surgery, Department of General Surgery,

The Second Hospital of Dalian Medical University, No.467 Zhongshan Road, Dalian, 116021, China

Tel: +860411-86110146

Fax: +860411-84671291

E-mail: sdgdoctor@163.com

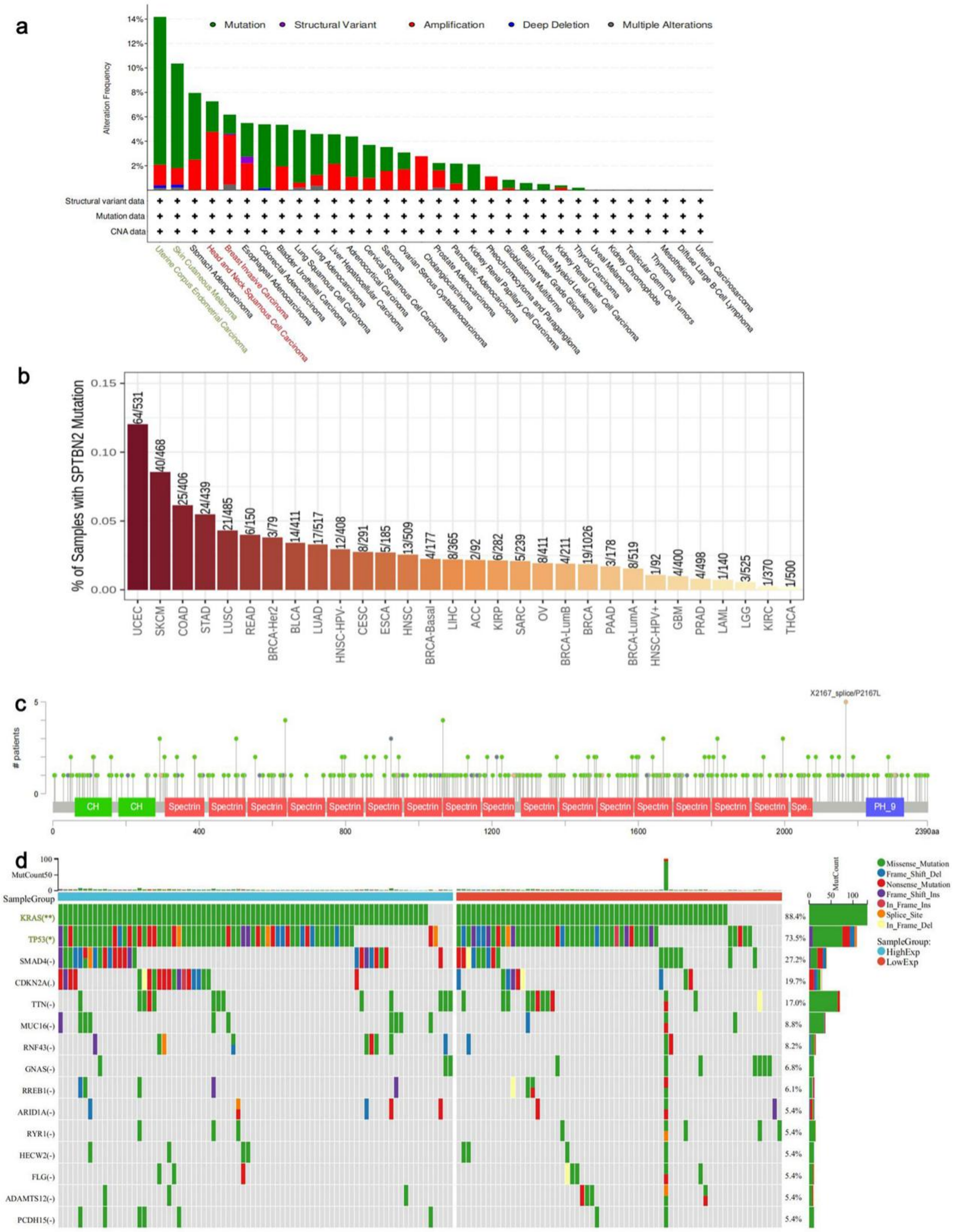

Supplemental Figure 1. Genetic alterations. (a) The cBioPortal database provides an overview of genomic alterations of the SPTBN2. (b) Mutation rates of SPTBN2 in various tumors via TIMER portal. (c) SPTBN2 mutation site. (d) Somatic mutations in SPTBN2 high and low expression groups. * $p < 0.05$, ** $p < 0.01$, *** $p < 0.001$, **** $p < 0.0001$

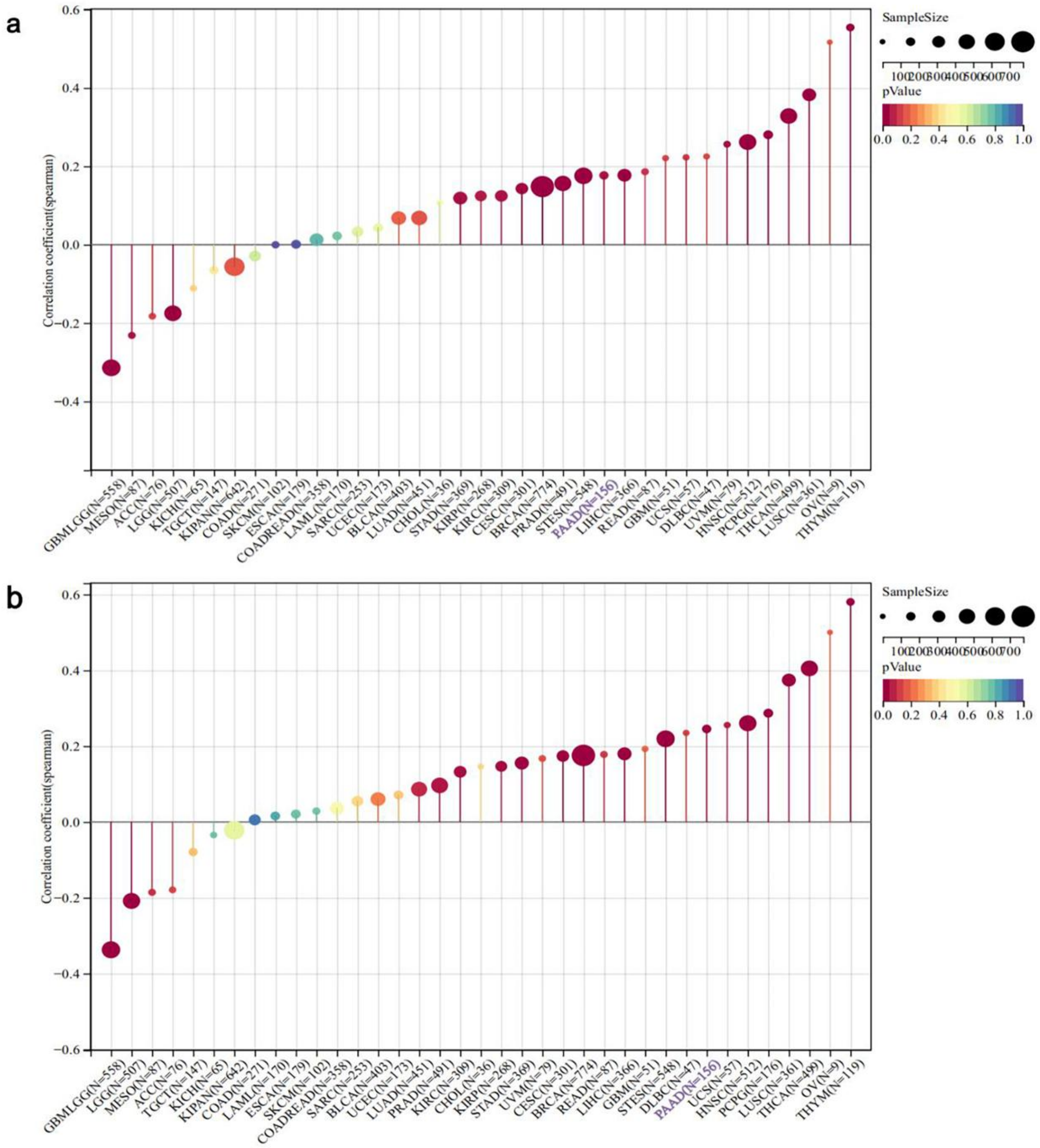

Supplemental Figure 2. SPTBN2 and tumor stemness. (a) Relationship between SPTBN2 and DNAss. (b) Relationship between SPTBN2 and EREG-METHss.

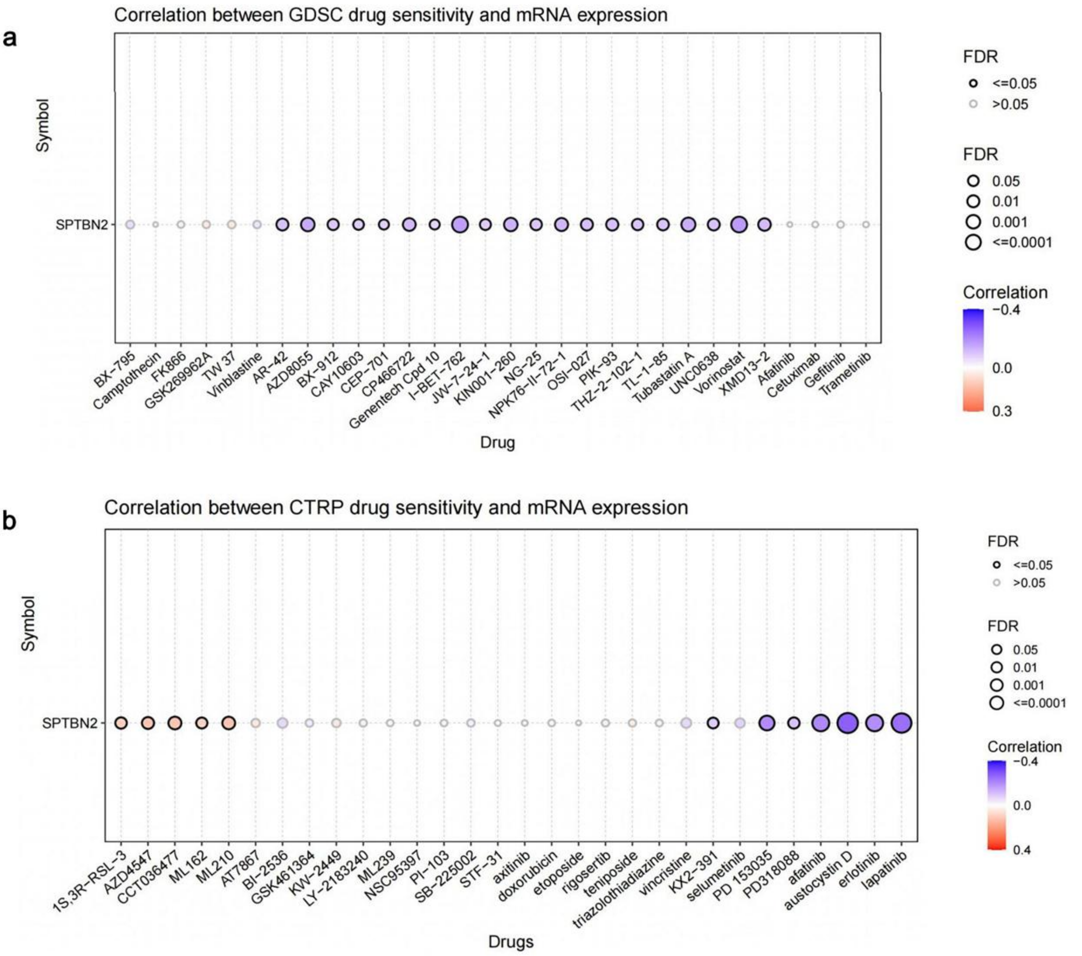

Supplemental Figure 3. Prediction of drug sensitivity based on SPTBN2 expression. (a) Predictive drugs based on the SPTBN2 expression from the GDSC datasets. (b) Predictive drugs based on the SPTBN2 expression from the CTRP datasets.
